# Talaromycosis in a Lung Cancer Patient: A Rare Case

**DOI:** 10.7759/cureus.10615

**Published:** 2020-09-23

**Authors:** Rosario Ching-López, Sara Rodríguez Pavón

**Affiliations:** 1 Radiation Oncology, Hospital Universitario Virgen de las Nieves, Granada, ESP

**Keywords:** non-small cell lung cancer, penicilliosis, fungemia, talaromycosis, emerging infectious diseases

## Abstract

Emergent fungal infections are rare conditions that frequently cause death. Talaromycosis is a fungal infection caused by *Talaromyces* sp. that is predominantly prevalent in patients with acquired immunodeficiency syndrome caused by human immunodeficiency virus infection, but in recent years we have noticed increasing reports of cases in people with other underlying conditions. We report a case of talaromycosis in a Stage IV non-small cell lung cancer female patient undergoing whole brain radiation therapy who presented to us with increasing dyspnea, cough and fever. The diagnosis was based on sputum and blood cultures, and even though our patient received anti-fungal treatment, the outcome was fatal. This case shows that a high index of suspicion could be essential for such a highly lethal but potentially treatable fungal infection.

## Introduction

Talaromycosis, previously known as penicilliosis, is a fungal infection caused by *Talaromyces* sp. This is a highly opportunistic thermally dimorphic fungal pathogen, and was first isolated in 1956 in Vietnam from the hepatic lesions of a bamboo rat (*Rhizomys sinensis*) [1,2]. We owe its description to Segretain (1959), who was himself the first human case reported as a consequence of an accidental inoculation injury from a contaminated needle [3]. It was not until 1973 when Di Salvo et al. described the first naturally acquired infection in a patient, and it was considered a rarity until the spread of the human immunodeficiency virus/acquired immunodeficiency syndrome (HIV/AIDS) pandemic in Southeast Asia in 1988, a region where this pathogen is now endemic [3,4].

However, over the past years we have noticed increasingly reported cases of infections due to *T. marneffei*, the etiological agent of talaromycosis, in non-HIV-infected patients who were affected with other immunocompromising conditions [3]. There are numerous potential reasons that could have led to this change in the epidemiology of talaromycosis, for example, improved treatment of HIV infection with highly active antiretroviral therapy and control of the HIV/AIDS epidemic, increased use of immune suppressive medications, better diagnostic tests, widened disease recognition, as well as global factors such as migration and travel increase [5]. Regardless of the cause, clinicians should be familiar with the changing epidemiology and clinical management of talaromycosis in potential hosts since an early use of antifungals is critical for improving the prognosis [6-8]. Herein, we report the case of an HIV-negative female patient who presented with talaromycosis coexisting with Stage IV primary non-small cell lung cancer (NSCLC). To the extent of our knowledge and literature review, this hasn't been reported before in Europe.

## Case presentation

**Figure 1:**
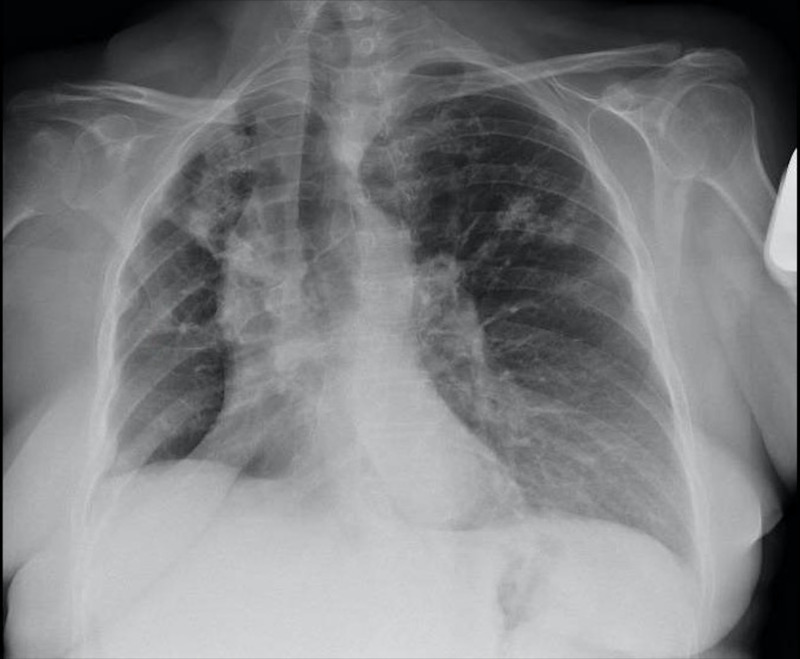
Chest X-ray image showing enlarged hilar shadow and interstitial infiltrates in the right upper and middle lobes

Our patient was a 56-year-old woman with a past medical history of chronic obstructive pulmonary disease (COPD), hypertension, and diabetes mellitus, and was diagnosed with NSCLC (initially Stage IIIB) 26 months ago. She underwent a right upper lobectomy followed by adjuvant chemotherapy with cisplatin-vinorelbine. After six months, the patient developed a local recurrence for which she underwent another extended resection. Within five months after, she experienced another mediastinal relapse according to the computed tomography (CT) and positron emission tomography (PET)-CT findings, and completed 30 sessions of radiotherapy (60 Gy) with concurrent administration of Taxol/carboplatin for six weeks. Two months later (26 months from diagnosis), she experienced a left-sided sudden hemiparesis and imaging revealed multiple brain metastases (Stage IV), for which she was given high-dose corticosteroids and was scheduled to receive whole brain radiotherapy (WBRT). After her second treatment session, she was admitted to our hospital with complaints of 38.1ºC fever, dyspnea, and cough. The patient had an Eastern Cooperative Oncology Group (ECOG) performance status of 2 and was hemodynamically stable. Oxygen saturation was 93% without supplementary oxygen. Physical examination showed diffuse crackles and rhonchi in all lung fields. Analytic studies (biochemistry and blood count) did not present significant findings, whereas the chest X-ray showed enlarged hilar shadow and airspace infiltrates in the right upper and middle lobes without evidence of pleural effusion or pneumothorax (Figure 1).

We began an antibiotic treatment with intravenous piperacillin-tazobactam (4 g/6 h). However, 24 h after admission, she continued to have fever (38.3ºC), and thus linezolid (600 mg/12 h) was added to the empirical treatment. In addition, pneumococcal and *Legionella* urinary antigen tests and bacilloscopy were carried out and the results were inconclusive. Nevertheless, serum galactomannan antigen test was positive and the fungal cultures sent from her sputum and blood yielded *Talaromyces* sp. after four days of incubation. The intravenous treatment was then optimized with liposomal amphotericin B 1800 mg/day while premedicating with normal saline and paracetamol. During the following 72 h, the patient remained afebrile, but her condition worsened and her oxygen saturation dropped to 87% (with supplementary oxygen at 2 lpm), requiring contribution with 40% oxygen to maintain saturation above the threshold of normality (>92%). Additionally, she developed moderate pancytopenia, her lab tests results being significant for hemoglobin of 9 g/dl, a white blood cell count of 800 cells/mm^3^, a neutrophil cell count of 570 cells/mm^3^, and thrombocytopenia with 54,000 platelets/µl. Her general status continued to worsen rapidly requiring 60% oxygen to maintain oxygen saturation and, despite targeted treatment, she developed shock and multiorgan failure. She eventually passed away six hours later.

## Discussion

Disseminated talaromycosis has been traditionally related to HIV-infected individuals residing in or traveling from endemic areas. However, an increasing number of cases have been reported in patients with other immunosuppressive conditions such as organ transplants or use of immunotherapy [3,9]. In fact, previous studies have proven that the main underlying diseases related to talaromycosis these days are not only hematological but also solid tumors [10-12], which is the case in our patient. This infection is characterized by the fungal invasion of multiple body systems, which may result in fever, cough, skin papules, and thoracalgia. In nonendemic areas, its low incidence and a lack of familiarity with its clinical manifestations can definitely contribute to the diagnostic difficulties [3,13], and given the aggressiveness of this pathogen, it is essential to include this etiology as a differential diagnosis in patients at risk. Based on the sputum and blood cultures, talaromycosis was diagnosed definitely in our patient five days after her admission; our literature review did not show any such previously reported cases in lung cancer patients in Europe.

The recent cycles of chemotherapy and the chronic use of corticosteroids at high doses most likely predisposed our patient to disseminated *T. marneffei* infection, rather than the cancer itself [1].

The lethality of this infection is informed to be 91.3% without early diagnosis and optimal treatment [1], whereas for properly treated non-HIV-infected patients, an estimated mortality rate of 33% can be deduced from a review of all published reports [3].

## Conclusions

In this study, treatment with liposomal amphotericin B was started five days after admission to hospital, but our patient died three days later. With the increasing use of immunosuppressive agents, it is mandatory for the healthcare community to remain vigilant against the reactivation of emerging fungal infections.
